# Oxidative pathways of apo, partially, and fully Zn(II)‐ and Cd(II)‐metalated human metallothionein‐3 are dominated by disulfide bond formation

**DOI:** 10.1111/febs.17333

**Published:** 2024-12-01

**Authors:** Amelia T. Yuan, Martin J. Stillman

**Affiliations:** ^1^ Department of Chemistry University of Western Ontario London Canada

**Keywords:** electrospray ionization mass spectrometry, metalloprotein, oxidative pathways, oxidative rates, reactive oxygen species

## Abstract

Oxidative stress is a key component of many diseases, including neurodegenerative diseases such as Alzheimer's disease. Reactive oxygen species (ROS) such as hydrogen peroxide and nitric oxide lead to disease progression by binding to proteins and causing their dysregulation. Metallothionein‐3 (MT3), a cysteine‐rich brain‐located metalloprotein, has been proposed to be a key player in controlling oxidative stress in the central nervous system. We report data from a combination of electrospray ionization mass spectrometry (ESI‐MS), ultraviolet (UV)‐visible absorption spectroscopy, and circular dichroism spectroscopy that identify the oxidation pathway of MT3 fully bound to endogenous Zn(II) or exogenous Cd(II) together with the partially metalated species. We characterize the intermediate species formed during the oxidation of MT3, which is dominated by disulfide bond formation. We report the rates of oxidation. For both fully and partially metalated MT3, MT3 is oxidized at 5 to 10 times the rate of MT1, a similar but kidney‐expressed isoform of MT. As oxidation progresses, MT3 follows a domain‐specific demetallation pathway when it is fully metalated, and a domain‐independent pathway when partially metalated. This suggests the presence of a significant susceptibility toward oxidation when MT3 is partially metalated, and, therefore, a possible protective role of Zn(II) when fully metalated. With the evidence for the rapid oxidation rate, our data support the proposals of MT3 as a key antioxidant in physiology.

## Graphical abstract

The electrospray ionization mass spectrometric experiments reported in this study describe the resistance to the oxidation of the cysteines of metallothionein‐3 to form disulfide bonds by the presence of differing stoichiometries of cadmium and zinc. The rate of oxidation of the cysteines in metallothionein‐3 was determined following addition of a single aliquot of the oxidant. The mass of the protein decreased by 2 Da for every disulfide bond formed.

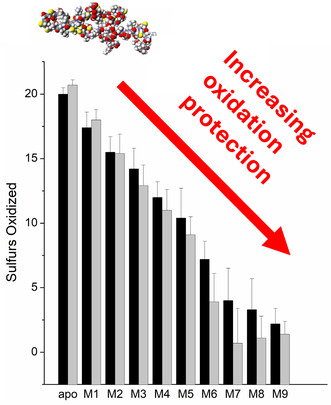

**Abbreviations**
CD: circular dichroism
ESI‐MS: electrospray ionization mass spectrometry
IPTG: isopropyl β‐d‐1‐thiogalactopyranoside
LMCT: ligand to metal charge transfer
MT1: metallothionein‐1
MT3: metallothionein‐3
NMR: nuclear magnetic resonance
ROS: reactive oxygen species
UV: ultraviolet

## Introduction

Reactive oxygen species (ROS) are the basis of many diseases in humans. ROS are produced through natural processes such as cellular respiration and exist in many forms, from hydrogen peroxide (H_2_O_2_) to nitric oxide (NO) [1, 2]. However, when produced in excess or when introduced from exogenous factors such as UV radiation, ROS can be harmful. ROS have the ability to cause DNA damage, resulting in carcinogenic mutations [2, 3, 4, 5]. ROS can also impact the structures and functions of lipids and proteins, which can impact cancer progression through signaling pathway breakdown [5, 6]. Moreover, ROS damage can result in neurological diseases [4].

Neurodegenerative diseases can be greatly impacted by oxidative stress as the brain consumes 20% of the oxygen supplied to the body [7, 8]. This is further exacerbated by metals which contribute to the progression of neurodegeneration [9, 10, 11, 12]. Cysteine‐rich antioxidant proteins may sequester ROS and in turn, form disulfide bonds to neutralize ROS [13, 14, 15, 16].

**Fig. 1:**
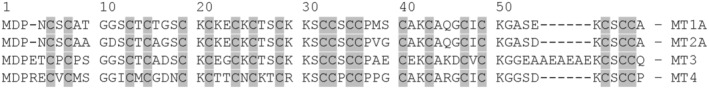
Amino acid sequences of human metallothioneins [60]. The accession numbers on UniProt are as follows: P04731 (MT1a), P02795 (MT2a), P25713 (MT3), and P47944 (MT4). Cysteines are highlighted in gray.

Mammalian metallothioneins (MTs) are small peptides with 20 conserved cysteines that can serve as antioxidants with greater effectiveness than GSH, due to a large number of cysteines available [17]. There are four isoforms of MTs, some of which are more frequently reported as redox agents than others. The amino acid sequences of Isoforms 1 through 4 are shown in Fig. 1, where we see the conservation of all 20 cysteines. Isoforms 1 and 2 are predominantly expressed in the kidneys and liver, respectively [18]. Isoform 3 (MT3) is expressed in the central nervous system and was thought to be more connected to the redox activity [19]. MT3 is induced by hypoxia‐inducible factor (HIF‐1), the key transcription factor activated in times of hypoxia, which often results in inflammatory responses that generate ROS [20, 21, 22]. MT3 has also been studied in hyperoxic environments, where it was shown to prevent neuron cell death through the scavenging of ROS [23, 24, 25]. In addition, as a Cu(I) binding protein, it has been reported that MT3 will scavenge oxidative Cu(II) in order to protect against Cu(II)‐exacerbated amyloid beta plaques of Alzheimer's disease [26, 27, 28].

When fully metal‐bound, MTs form distinct structures, including a Zn/Cd_4_S_11_ cluster in the α domain and a Zn/Cd_3_S_9_ cluster in the β domain [29, 30, 31, 32, 33, 34, 35, 36, 37]. These clusters are also induced under stress conditions, such as the case with acidic pH or protein–protein interactions [38, 39]. Although it is important to study Cu(I) bound MT3, the complexity of Cu(I)‐MT3, especially in combination with Zn(II), requires first the evaluation of Zn(II) and Cd(II) MT3 to set the foundation for understanding Cu(I)‐MT3 or mixed Cu‐Zn‐MT3 oxidation [40, 41].

In a recent study of the oxidation of MT1 exposed to hydrogen peroxide, it was found that oxidation occurs with the sequential loss of metal ions as a function of disulfide bond formation [42]. The only study in the literature on the oxidation of MT3 is the analysis of Cd_7_MT3 exposure to nitric oxide, monitored by NMR methods [43]. However, no specific details were reported.

In this study, we describe the oxidation mechanism of fully and partially Zn(II) and Cd(II) metalated MT3. It is important to determine the specific pathways of oxidation in MT3 compared with the liver and kidney isoforms (MT1 and MT2, respectively) because of the presence of its unique TCPCP motif in the β domain and hexapeptide acidic loop insert in the α domain, and in addition, its biologically specific function in the central nervous system [44, 45, 46]. We find that oxidation of apo‐MT3 rapidly forms 10 disulfide bonds when exposed to excess hydrogen peroxide. When both fully and partially metalated with Zn(II)‐ or Cd(II), MT3 is oxidized sequentially, meaning that disulfide bond formation takes place in measurable single steps. The observed rates are significantly faster than those observed previously for MT1 [42]. In addition, oxidation induces the Cd_4_MT3 cluster formation before complete oxidation of the peptide has taken place. This could again be a protective mechanism from oxidation due to the cluster stability. For Zn_7_MT3, the oxidation pathway involves a smaller fraction of Zn_4_MT3 cluster formation during oxidation. Significantly, oxidation of the two methionine's in the MT3 sequence only takes place when the 20 reduced cysteines have been oxidized. Using electrospray ionization mass spectrometry (ESI‐MS), circular dichroism (CD), and UV–visible absorption spectroscopy methods, we provide evidence for the increased susceptibility of MT3 to oxidation and, thus, support its role as an effective antioxidant.

## Results and Discussion

### Oxidation of apo‐MT3 by hydrogen peroxide

**Fig. 2:**
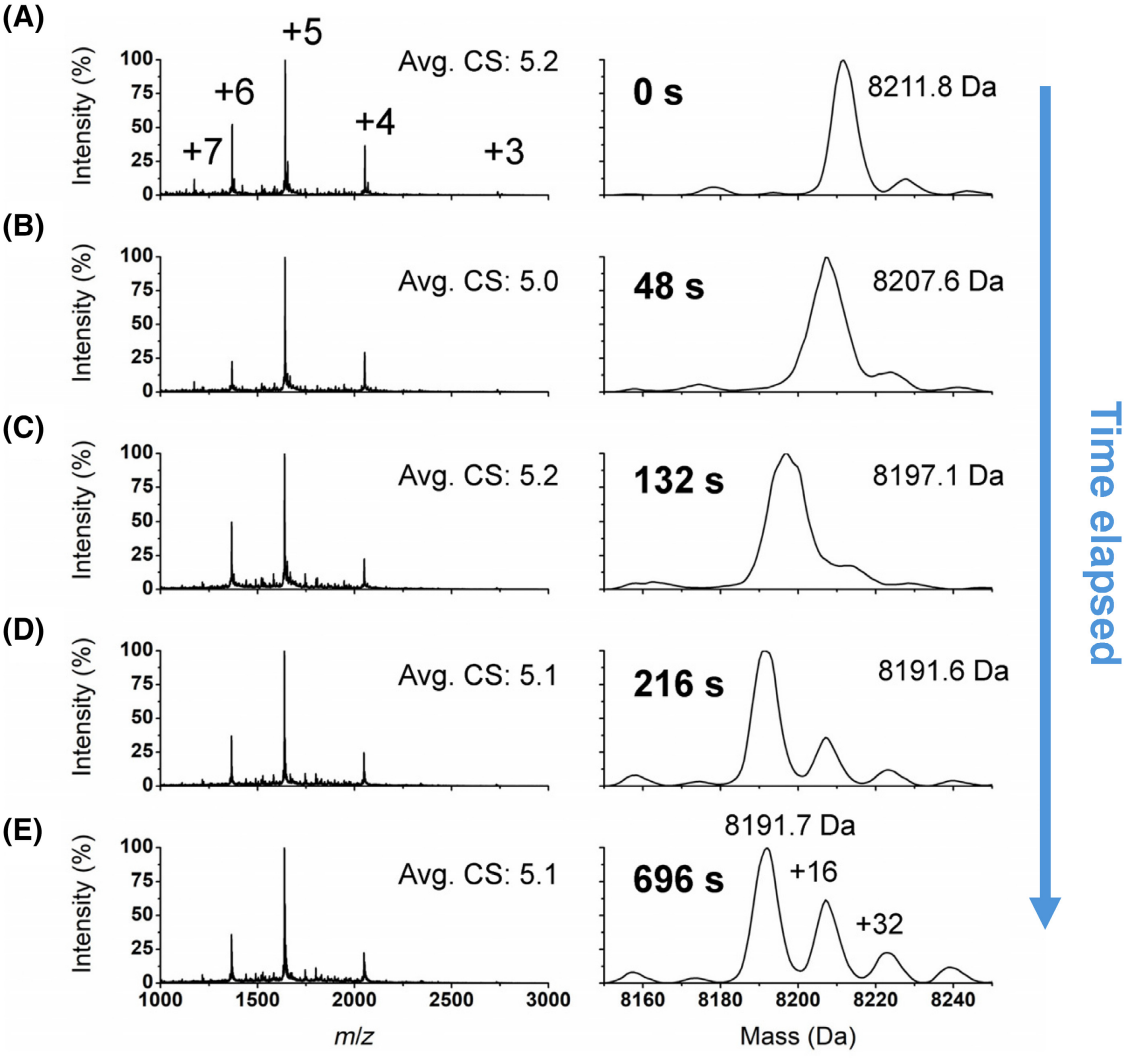
Oxidation of apo‐MT3 at pH 7.4. A 50 μm apo‐MT3 sample was prepared in 20 mm ammonium formate at pH 7.4 and 150 molar equivalents of H_2_O_2_ was added. (A–E) A sample of electrospray ionization (ESI)‐mass spectra recorded during the time‐dependent oxidation reaction with apo‐MT3. The left panels are the charge state spectra with the charge states (CS) labeled in Panel A, which applies to the panels underneath. The average charge state for each spectrum is calculated by the relative intensities of each peak and labeled. The right panels are the corresponding deconvoluted spectra, with the apo‐MT3 peak labeled. Fully reduced apo‐MT3 is labeled in Panel A. The data presented represents *n* = 1 of 3 repeated experiments.

Figure 2 shows the ESI‐MS data recorded as a function of time following the addition of 150 molar equivalents of H_2_O_2_ to a solution of 50 μm apo‐MT3 at physiological pH. Complete oxidation corresponding to a 20 Da shift due to loss of 20 cysteinyl thiolate protons with the formation of 10 cysteine disulfide bonds takes place by 216 s (Fig. 2D). The charge state spectra on the left panels show that the average charge state remains essentially unchanged between the reduced and oxidized apo‐MT3. The data show that the reaction takes approximately 200 s to fully oxidize the apo‐MT3, which is identified by a mass centered on 8919.6 Da. Therefore, even with 150 molar equivalents of H_2_O_2_ added, there is still some protection of cysteines from oxidation, as the reaction is not instantaneous. By using a sample of apo‐MT3 with already a small fraction of one methionine oxidized at +16 Da (Fig. 2A), we can determine the pathway of oxidation as being initially entirely reduced cysteine because the methionine oxidized fraction of protein only increases in relative abundance once 20 cysteines have been oxidized (Fig. 2E).

Following the complete oxidation of the reduced cysteines, peaks increasing by 16 Da appear (Fig. 2E), that we attribute to methionine sulfoxide formation, as noted previously for MT1 [42]. For MT3, there are only two methionine residues; therefore, only +16 and +32 Da peaks appear. There is no evidence of increased sulfonic acid formation, as no additional peaks of +16 Da were noted. Even though cysteine sulfenic, sulfinic, and sulfonic acid had been reported *in vivo* in the literature, it appears that disulfide bond formation is preferable over these other products of oxidation [47]. It is important to note, however, that methionine sulfoxide is formed after disulfide bond formation; therefore, cysteine sulfoxide formation may not occur due to the rapid kinetics of disulfide bond formation.

### Oxidation pathway of Cd_7_MT3

**Fig. 3:**
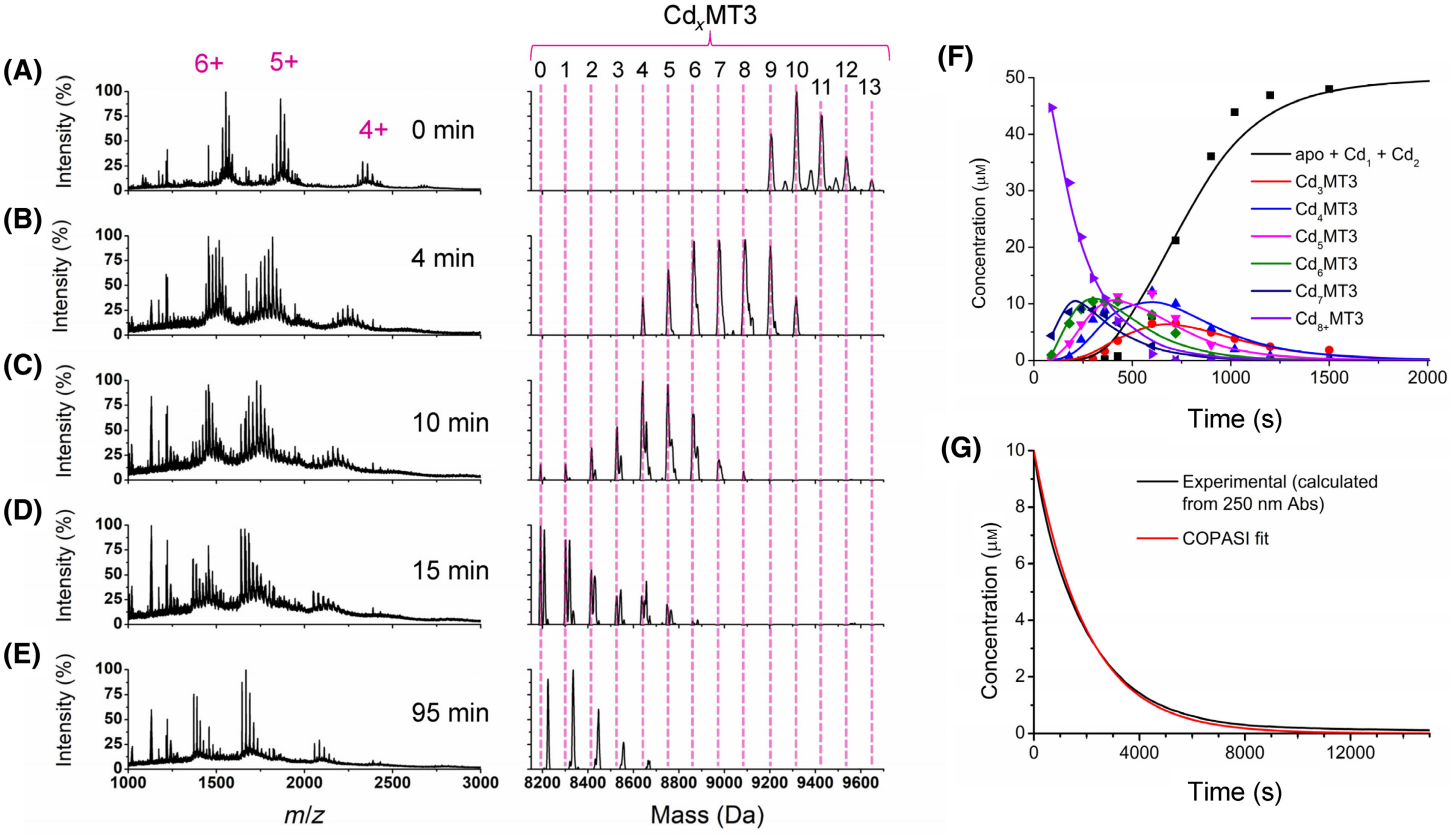
Reaction of Cd_7_MT3 with 150 molar equivalents of H_2_O_2_ at pH 7.4 monitored over time using ESI‐MS and UV–visible absorption spectroscopy. The concentration of Cd_7_MT3 used for the ESI‐mass spectral analysis was 50 μm, whereas the concentration used for the UV–visible absorption spectroscopy was 10 μm. The initial solution was made by adding approximately 7 molar equivalents of Cd(II) to apo‐MT3, based on UV–visible absorption spectrum. (A–E) Selected mass spectral data of the reaction at time points (A) 0 min, (B), 4 min, (C) 10 min, (D), 15 min, and (E) 95 min. The charges state spectra are the left panels. The charge states labeled in Panel A apply to the panels below. The corresponding deconvoluted mass spectra are shown in the right panels. The masses for each Cd_*x*_MT3 species (*x* = 0–13) are labeled. Note that the masses of the peaks may not exactly match those calculated for the Cd_*x*_MT3 species due to the addition of oxygen (+16 Da) as well as the oxidation of cysteines to disulfide bonds. (F) Corresponding concentrations of each Cd_*x*_MT3 species as a function of time (points) extracted from the ESI‐mass spectral data with fitted speciation calculated by copasi (solid lines). (G) Kinetic trace of the absorption at 250 nm for a 10 μm solution of Cd_7_MT3 (this solution is 5× more dilute than the solution used in the ESI‐MS) as a function of time (black line) with fitted kinetic trace calculated by copasi for the ESI‐MS data adjusted for the 10 μm concentration of Cd_7_MT3 (red line). The data presented represents *n* = 1 of 3 repeated experiments.

Fully metalated Cd_7_MT3 was oxidized with hydrogen peroxide at physiological pH (Fig. 3). The ESI‐MS data show that the initial reaction induces the formation of the more highly metalated species such as Cd_8–13_MT3. We interpret this as the formation of adducts, or Cd(II) ions that are not fully coordinated by four cysteines but nonspecifically bound to the protein (Fig. 3A). The fully metalated, two‐domain Cd‐MT3, however, exhibits higher overall charge states than apo‐MT3. The apo‐MT3 charge state spectrum is dominated by the +5 charge, whereas for Cd‐MT3, the +6 charge state is approximately equal in intensity to the +5 charge state (Fig. 3A). The charge state distribution shifts to an overall lower average with as oxidation progresses (Fig. 3E), where it closely resembles the oxidized apo‐MT3 in Fig. 1. It follows that the surface area represented by the charge state spectra shifts from one of higher surface area with the Cd‐bound MT3 to lower surface area with the loss of Cd(II); therefore, disulfide bond formation appears to stabilize a more compact structure for the MT3. This is consistent with the report of disulfide bond formation in MT2 resulting in a shorter drift time and collision cross‐section through ion mobility MS studies [48].

**Scheme 1:**
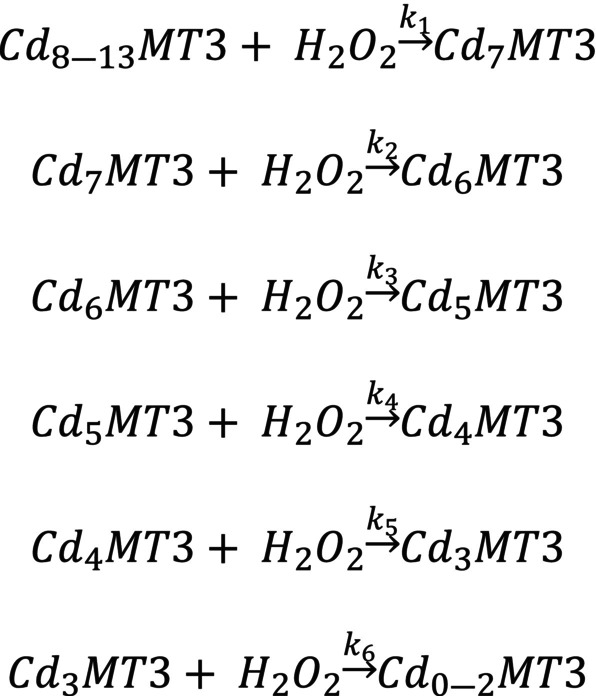
The sequential demetallation pathway of Cd_7_MT3 as a result of oxidation with H_2_O_2_. The rate constants for each oxidation and demetallation event is labelled as *k*_1–6_.

The pathway of oxidation for Cd_7_MT3 can be represented by Scheme 1, which is the sequential demetallation pathway of Cd_7_MT3 as a result of oxidation with H_2_O_2_. The rate constants for each oxidation and demetallation event are labeled as *k*_1–6_.

The mass changes shown in Fig. 3A–E indicate that each oxidation step involves approximately one disulfide bond formation. Table 1 shows the calculated mass of the reduced Cd_*x*_MT3, the experimental mass of Cd_*x*_MT3, and the oxidized cysteines as well as the predicted disulfide bonds. Please note that two oxidized cysteines will be incorporated into one disulfide bond, and therefore, 2 Da loss of the hydrogens on two cysteines constitute a disulfide bond formation. The speciation of the Cd_*x*_MT3 species can be mapped as a function of time (Fig. 3F) where we see the stepwise demetallation of the Cd_*x*_MT3 species. Cd_4_MT3 appears to persist in the speciation diagram for longer than most Cd_*x*_MT3 species (from approximately 250 to 1500 s); therefore, it is probable that this may be a stable species more resistant to oxidation. However, the Cd_*x*_MT3 species are relatively, normally distributed (Fig. 3A–E) with no clear indication of a cooperatively formed species. This lack of clear cooperative formation of the Cd_4_ cluster in the MT3 α domain has been reported as a property of MT3 during metalation [49]. This is further supported by the relatively weak ^113^Cd NMR resonances and the requirement of high concentrations of MT3 in order to obtain ^113^Cd NMR resonances, as well as multiple reports of the flexibility and fluxionality of the MT3 peptide backbone [32, 33, 45, 50]. In addition, acidification titrations have indicated that there is no clear cooperative release of metals indicative of separate release of the metals in the α and β domains for MT3, as was seen for MT2 [51].

**Table 1:** The experimental mass values for Cd_*x*_MT3. The number of cysteines oxidized is calculated using the difference between the theoretical and experimental masses, as each cysteine oxidized corresponds to 1 Da lost for the 1 H lost.

Species	Mass experimental reduced (Da)	Mass experimental (Da)	Cysteines oxidized (Mass_Calc_ − Mass_exp_)	Estimated S–S bonds
Apo‐MT3	8212.0	8192.0	20.0	10
Cd_1_MT3	8322.4	8305.0	17.4	9
Cd_2_MT3	8432.8	8417.3	15.5	8
Cd_3_MT3	8543.5	8529.3	14.2	7
Cd_4_MT3	8654.2	8642.2	12.0	6
Cd_5_MT3	8765.3	8754.9	10.4	5
Cd_6_MT3	8874.3	8867.1	7.2	4
Cd_7_MT3	8986.1	8982.1	4.0	2
Cd_8_MT3	9095.6	9092.3	3.3	2
Cd_9_MT3	9206.4	9204.2	2.2	1

The last step of the oxidation of Cd_7_MT3 involves Cd_3_MT3 to Cd_0–2_MT3, which was combined into one step due to the incomplete demetallation and subsequent low re‐metalation of apo‐MT3 to species Cd_1–2_MT3. The mass differences indicate that these Cd(II) ions are adducts, as an average of 20 cysteines are already oxidized at this point.

It is also important to note that the mass spectra show increases in mass of +16 and +32 Da (Fig. 3C–E) similar to the spectra recorded for apo‐MT3 oxidation in Fig. 2. This is consistent with the explanation that methionine sulfoxide is forming during the latter steps of oxidation, and mostly after the cysteines of MT3 have been fully oxidized.

Oxidation occurs at a higher rate overall and with less cooperativity in the formation of the Cd_4_ cluster compared to other MTs. The reaction is mostly complete by 20 min, as by 15 min (Fig. 3D), only Cd_0–4_MT3 exist, approximately half the time noted for other MTs.

The rate constants for each oxidation step were calculated using the copasi software by fitting the experimental data in Fig. 3F. The fitted traces of each oxidation step are shown by the solid lines in Fig. 3F. The rate constants (*k*_1–6_) are as follows for the reactions shown in Scheme 1: 1.2 ± 0.6 m ^−1^·s^−1^, 2.2 ± 0.6 m ^−1^·s^−1^, 0.7 ± 0.4 m ^−1^·s^−1^, 1.3 ± 0.3 m ^−1^·s^−1^, 1.5 ± 0.4 m ^−1^·s^−1^, and 2.0 ± 0.3 m ^−1^·s^−1^. The oxidation reaction was repeated three separate times to obtain the standard deviation reported.

The oxidation of Cd_7_MT3 was also carried out using the 250 nm S‐Cd charge transfer (ligand to metal charge transfer—LMCT) band to monitor the Cd‐loading as a function of time following the addition of the same ratio of H_2_O_2_ (Fig. 3G). In this experiment, however, 10 μm protein was used to keep absorption at 250 nm within acceptable range for sensitive and accurate detection by the spectrometer. A simulated reaction trace of the overall loss of Cd was then calculated based on the six rate constants of the ESI‐mass spectral data (Fig. 3F). The experimental data from the UV–visible absorption measurement and the simulated kinetic trace based on the analysis of the ESI‐mass spectral data are essentially the same, taking into account the 50 μm ESI‐MS concentration compared with the 10 μm absorption concentration. We conclude that the rates determined from the ESI‐mass spectral data represent the rates of oxidation in the optical cuvette.

The rate constants obtained for MT3 oxidation under the same conditions are between 5 and 10 faster than the rates reported for MT1A. This is a significant difference in oxidation kinetics, especially with minimal changes in protein sequence, with a TCPCP motif in the β domain and a 6‐amino acid length acidic loop insert in the α domain [44, 45]. This may be interpreted as a result of the fluxionality of the MT3, as mentioned in the past [32, 33, 51, 52]. In addition, the binding affinity for Cd(II) may also be lower than other MTs, as noted by the equilibrium of Cd(II) ions preferentially binding to MT2 before MT3 [49]. In addition, the flexibility of the MT3 may also be a factor, which was proposed to allow for additional sites of metal binding [53].

Due to the mostly noncooperative pattern of oxidation and metal loss when MT3 is oxidized, each metal is lost sequentially and can represented by one reaction step in the pathway, and thus, a single rate constant. Cd_7_MT3 oxidizes first to Cd_6_MT3 before Cd_6_MT3 oxidizes to Cd_5_MT3, indicating that the continued presence of the bridging thiolate network is not cooperatively disrupted by oxidation with H_2_O_2_. This further supports the noncooperative nature of metal binding to MT3 [49]. These observations allow us to suggest that the rate of oxidation is faster due to the loss of the protective mechanism of clusters.

### Oxidation pathway of Zn_7_MT3

**Fig. 4:**
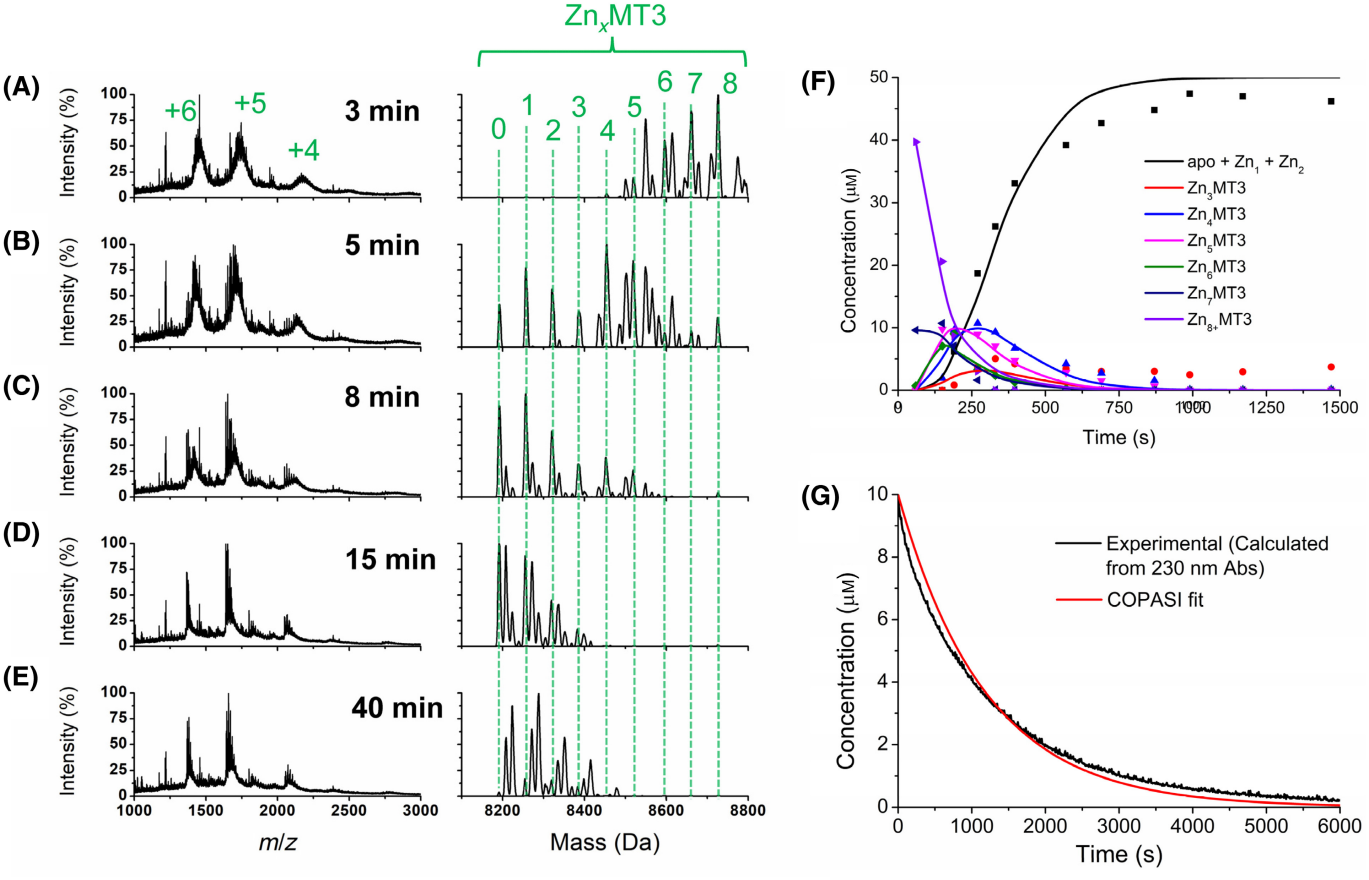
Reaction of Zn_7_MT3 with 150 molar equivalents of H_2_O_2_ at pH 7.4 monitored over time using ESI‐MS and UV–visible absorption spectroscopy. The concentration of Zn_7_MT3 used for the ESI‐mass spectral analysis was 50 μm, whereas the concentration used for the UV–visible absorption spectroscopy was 10 μm. (A–E) Selected mass spectral data of the reaction at time points (A) 3 min, (B), 5 min, (C) 8 min, (D), 15 min, and (E) 40 min. The charges state spectra are the left panels. The charge states labeled in Panel A apply to the panels below. The corresponding deconvoluted mass spectra are shown in the right panels. The masses for each Zn_*x*_MT3 species (*x* = 0–8) are labeled. Note that the peaks may not exactly match that calculated for the Zn_*x*_MT3 species due to addition of oxygen (+16 Da) as well as oxidation of cysteines to disulfide bonds. (F) Corresponding concentrations of each Zn_*x*_MT3 species as a function of time (points) extracted from the ESI‐mass spectral data with fitted speciation calculated by copasi (solid lines). (G) Kinetic trace of the absorption at 230 nm for a 10 μm solution of Zn_7_MT3 (this solution is 5× more dilute than the solution used in the ESI‐MS) as a function of time (black line) with fitted kinetic trace calculated by copasi for the ESI‐MS data adjusted for the 10 μm concentration of Zn_7_MT3 (red line). The data presented represents *n* = 1 of 3 repeated experiments.

**Scheme 2:**
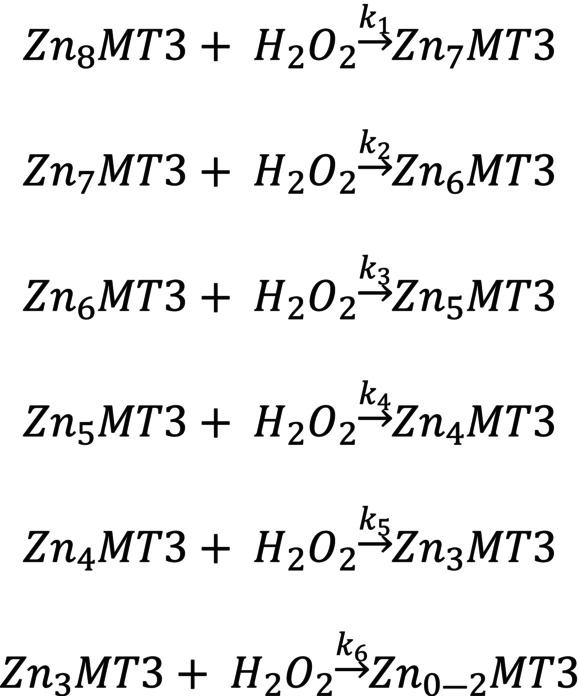
The sequential demetallation pathway of Zn_7_MT3 as a result of oxidation with H_2_O_2_. The rate constants for each oxidation and demetallation event is labelled as *k*_1–6_.

Fully metalated Zn_7_MT3 was oxidized with 150 molar equivalents of H_2_O_2_ and monitored using ESI‐MS and UV–visible absorption spectroscopy (Fig. 4). The average charge states of the Zn_7_MT3 and each of the Zn_*x*_MT3 species that formed during the oxidation reaction are essentially the same as for Cd_7_MT3, where the charge states with the greatest intensity are +6 and +5, with a shift toward +5 as oxidation proceeds (Figs 3A–E and 4A–E). Again, +16 Da peaks appear in the deconvoluted spectra as a result of the formation of methionine sulfoxide, notably in Fig. 4D,E. The demetallation itself occurs sequentially as found during the oxidation of Cd_7_MT3, including an initial shift to higher metalated states as a result of exposure to H_2_O_2_ (Fig. 4A). However, the presence of a persistent Zn_4_MT3 species representing the Zn_4_ cluster in the α domain is more apparent than in the Cd_4_ cluster (Fig. 4B). This Zn_4_ species, however, quickly disappears by 8 min (Fig. 4C), where the dominant species is now apo‐MT3. The experimental mass values of Zn_*x*_MT3 in both reduced and oxidized form are listed in Table 2, as well as the cysteines oxidized and the corresponding disulfide bonds formed. The pathway of demetallation of Zn_7_MT3 as a result of oxidation with H_2_O_2_ is summarized in Scheme 2, where the rate constants for each oxidation and demetallation event are labeled as *k*_1–6_.

**Table 2:** The experimental mass values for Zn_*x*_MT3. The number of cysteines oxidized is calculated using the difference between the theoretical and experimental masses, as each cysteine oxidized corresponds to 1 Da lost for the 1 H lost.

Species	Mass reduced (experimental)	Mass (experimental)	Cysteines oxidized (Mass_Calc_ − Mass_exp_)	Estimated S–S bonds
Apo‐MT3	8212.0	8191.3	20.7	10
Zn_1_MT3	8275.3	8257.3	18.0	9
Zn_2_MT3	8338.7	8323.3	15.4	8
Zn_3_MT3	8402.1	8389.2	12.9	6
Zn_4_MT3	8465.3	8454.3	11.0	6
Zn_5_MT3	8528.9	8519.8	9.1	5
Zn_6_MT3	8592.4	8588.5	3.9	2
Zn_7_MT3	8655.8	8655.1	0.7	0
Zn_8_MT3	8719.0	8717.9	1.1	0
Zn_9_MT3	8782.0	8780.6	1.4	0

Again, the emergence of each Zn_*x*_MT3 species can be plotted as a function of time in Fig. 4F (symbols). These experimental data indicate that the reaction is mostly complete at approximately 500 s, which is much faster than for Cd_7_MT3, where the reaction takes until 1000 s to be complete. These rate constants were calculated by fitting the experimentally determined speciation to the Scheme 2 model using copasi, where the rate constants (*k*_1–6_) calculated are as follows: 1.5 ± 0.7 m ^−1^·s^−1^, 2.9 ± 0.2 m ^−1^·s^−1^, 2.9 ± 0.8 m ^−1^·s^−1^, 1.8 ± 0.5 m ^−1^·s^−1^, 2.2 ± 0.2 m ^−1^·s^−1^, and 4.5 ± 1.8 m ^−1^·s^−1^. The large error noted for the last step of the reaction (*k*_6_) is high due to the rapid rate of reaction because of the difficulty in capturing this last reaction step by ESI‐MS methods. These rate constants were tested by transformation to create a simulated reaction trace of the overall demetallation reaction that would be observed at the 230 nm LMCT band and superimposed on the reaction trace that was experimentally determined absorbance at 230 nm for Zn(II) demetallation using H_2_O_2_ under the same conditions (Fig. 4G). These data show that the ESI‐mass spectral data follow the kinetic rate of oxidation as observed for solutions of Zn_7_MT3 exposed to 150 molar equivalents of H_2_O_2_ monitored by the 230 nm S‐Zn charge transfer absorption band. It is important to note, however, that 10 μm protein was used to keep absorption at 250 nm within acceptable range for sensitive and accurate detection by the spectrometer. The copasi fit in Fig. 4G was calculated using the obtained rate constants to demonstrate an average rate of Zn loss and thus oxidation. With the overall reaction trace in Fig. 4G, it is visibly faster than the oxidation reaction of Cd_7_MT3 (Fig. 3G), where the trace plateaus at approximately 4000 s for Zn_7_MT3 and 8000 s for Cd_7_MT3.

A comparison of the individual values of rate constants for each step of the oxidation reaction shows that these rate constants are approximately 2× larger than those for the Cd_7_MT3 reaction. This increase in oxidation susceptibility is likely due to the lower binding affinity of Zn(II) to MT3 which are approximately log(*K*_F_) = 11, and, therefore, provide less protection against oxidation of the cysteines [39, 54, 55]. In addition, the Zn cluster formation may also be less stable than in other MTs, as noted by the circular dichroism spectroscopy [53]. A clear derivative band centered at 230 nm, where the S‐Zn charge transfer occurs, is indicative of symmetry that occurs from the two pairs of Zn(II) in the Zn_4_ cluster of the α domain [56, 57]. This CD signature band is essentially absent for Zn‐MT3, where this derivative band is extremely weak and not nearly as well‐defined as seen for other MTs [53].

Comparison of the rate constants of MT1A with those of MT3, we find that the rates are again between 5 and 10 times faster for MT3 [42]. This is not surprising due to the increased rate of oxidation of the Cd_7_MT3; however, it is in contrast to what was reported by Chen *et al*. [58] It is unclear why MT3 reacts with greater efficiency than seen with MT1 in our studies compared to Chen *et al*., however, buffer conditions differed and their tracking of Zn(II) release was accomplished with ZINCON fluorescence. We note that, similar to Cd(II), Zn(II) does not fully release from the MT3 protein, as some Zn(II) remains as adducts even after 40 min (Fig. 3E). This may be the reason the amount of Zn(II) released due to oxidation by H_2_O_2_ as determined by ZINCON by Chen *et al*. is lower for MT3. However, the same study found that Zn(II) is more rapidly released from MT3 than MT1 and MT2 due to oxidation from S‐nitrosothiols, another biologically relevant oxidizing agent. The demetallation as a result of oxidation again occurs in a relatively normally distributed manner similar to MT1, where all Zn_*x*_MT3 species, where *x* = 0–8 is reported on the ESI‐mass spectral data [42].

### Cd_4_MT3 and Zn_4_MT3 clusters are formed during oxidation

**Fig. 5:**
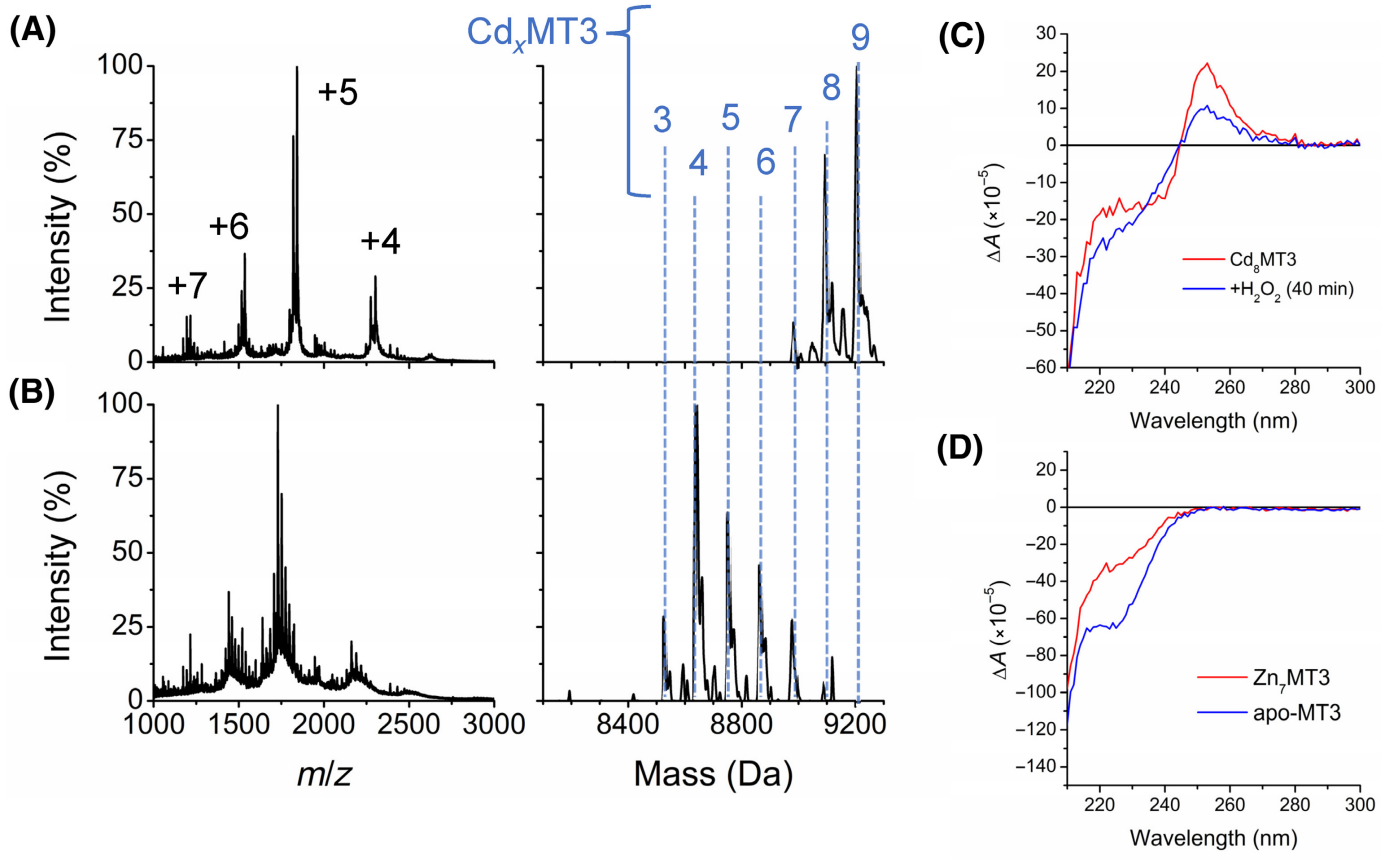
Circular dichroism spectra and corresponding mass spectral data of solutions of 25 μm Cd_7_MT3 and Zn_7_MT3. (A, B) ESI‐mass spectral data showing Cd_7_MT3 (A) and Cd_4_MT3 after 40 min of oxidation with 40 molar equivalents of H_2_O_2_ (B) with the charge state spectra on the left with labeled charge states and corresponding deconvoluted mass spectra on the right. The corresponding circular dichroism data are shown in (C) with the red trace corresponding to the sample measured in (A) and the blue trace corresponding to the sample measured in (B). (D) Circular dichroism spectra of Zn_7_MT3 with very weak derivative band signatures. Data of apo‐MT3 are shown for comparison purposes. The data presented represents *n* = 1 of 3 repeated experiments.

In order to probe the structures formed during oxidation, the ESI‐mass spectral and circular dichroism (CD) spectra data were recorded in parallel during the oxidation reaction of Cd_7_MT3 (Fig. 5A–C). The fully metalated Cd_7–8_MT3 was measured by ESI‐MS (Fig. 5A) together with the corresponding Cd_8_MT3 CD spectrum (red line in Fig. 5C). The reaction with 40 molar equivalents of H_2_O_2_ was then allowed to proceed for 40 min. The ESI‐mass spectral data and CD spectral data were recorded at this time (Fig. 5B,C blue line). Only 40 molar equivalents of H_2_O_2_ were used in order to slow down the reaction. When analyzing the CD spectrum, we observe a derivative band with a positive signal at 250 nm, a weak negative band at 220 nm, and a crossover point at 245 nm for both the initial Cd_8_MT3 and the solution 40 min after oxidation, meaning there is a fraction of the Cd_4_ clusters still intact in the solution. The Cd_4_ cluster, although not very apparent as a significant species in the ESI‐mass spectral data from Fig. 3, is still a major species in the oxidation pathway of Cd_7_MT3, as observed in the CD spectra in Fig. 5. The same experiment was attempted for Zn_7_MT3 oxidation; however, the low intensity of the derivative band in the CD spectrum representative of the Zn clusters (Zn_4_S_11_) in Zn_7_MT3 (red trace, Fig. 5D) showed little resolution between Zn_7_MT3 and other Zn_*x*_MT3 species. The CD spectrum for apo‐MT3 is shown for comparison in Fig. 5D. This is consistent with the weak derivative band observed in the CD spectrum of Zn‐MT3 reported previously [53].

### Partially metalated Cd‐MT3 reaction with hydrogen peroxide

**Fig. 6:**
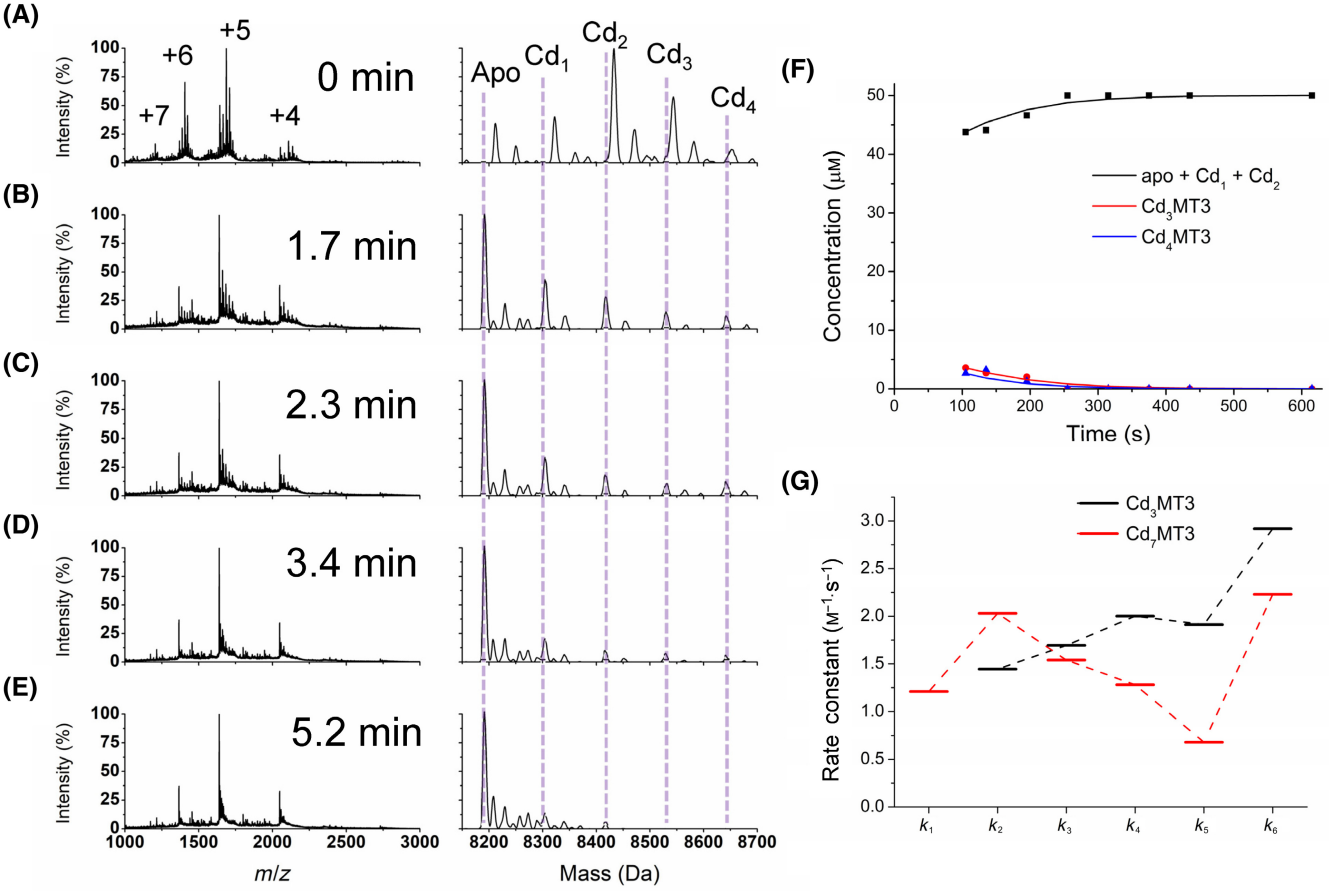
Reaction of Cd_3_MT3 with 150 molar equivalents of H_2_O_2_ at pH 7.4 monitored over time using ESI‐MS and UV–visible absorption spectroscopy. The concentration of Cd_3_MT3 used for ESI‐mass spectral analysis was 50 μm. (A–E) Selected mass spectral data of the reaction at time points (A) 0 min, (B), 1.7 min, (C) 2.3 min, (D), 3.4 min, and (E) 5.2 min. The charges state spectra are the left panels. The charge states labeled in Panel A apply to the panels below. The corresponding deconvoluted mass spectra are shown in the right panels. The masses for each Cd_*x*_MT3 species (*x* = 0–4) are labeled. Note that the peaks may not exactly match that calculated for the Cd_*x*_MT3 species due to addition of oxygen (+16 Da) as well as oxidation of cysteines to disulfide bonds. (F) Corresponding concentrations of each Cd_*x*_MT3 species as a function of time (points) extracted from the ESI‐mass spectral data with fitted speciation calculated by copasi (solid lines). (G) Rate constants (*k*) for each oxidation step outlined in Scheme 1 for Cd_3_MT3 (black) and Cd_7_MT3 (red). The data presented represents *n* = 1 of 3 repeated experiments.

Following the determination of the pathway of oxidation of fully metalated Cd_7_MT3 and Zn_7_MT3, partially metalated species were used to quantify their reaction with H_2_O_2_. In Fig. 6, the data for oxidation of partially metalated Cd‐MT3 are shown. An aliquot of 3 molar equivalents of Cd(II) was added to the apo‐MT3 and the charge state and deconvoluted spectra are shown in Fig. 6A–E. The expected masses of the oxidized species are labeled in Fig. 6A–E. The observed species in Fig. 6A have slightly higher masses due to the presence of the hydrogens on the reduced thiolates at this point. Figure 6B–E shows selected time points of oxidation after 150 molar equivalents of H_2_O_2_ are added. The charge states again show a shift from dominant charges of +5 and +6 to mainly +5 as oxidation occurs. The deconvoluted spectra show the fast demetallation process due to the oxidation, where apo‐MT3 becomes the dominant species in the spectra after only 1.7 min. The speciation as a function of time is shown in Fig. 6F, with the fitted copasi traces in solid lines and the points as experimentally determined speciation. The fitted rate constants are as follows (*k*_5–6_): 1.9 ± 0.4 m ^−1^·s^−1^, and 2.9 ± 0.9 m ^−1^·s^−1^. These rate constants, as well as the Cd_7_MT3 oxidation rate constants, are summarized in Fig. 6G. Please note that only time 100 s and beyond was used because the mixed solution of hydrogen peroxide and the MT3 solution did not reach the detector with an acceptable count per MT3 species. The time 0 s mass spectrum was taken before the H_2_O_2_ solution was added. Scheme 1 shows the reactions used, as the sequential demetallation pathway of Cd_7_MT3 as a result of oxidation with H_2_O_2_. The rate constants for each oxidation and demetallation event are labeled as *k*_1–6_.

The trend in the rates of oxidation is similar to the trend observed for Cd_7_MT3, where there are no intermediate species that are specifically favored, such as the formation of Cd_4_MT3 or apo‐MT3 that were seen with the cooperative metalation pathway [38, 39]. The rates of reaction, however, were extremely fast, and therefore, determining the presence of intermediates was difficult. Multiple replicates of the oxidation reaction yielded fitted rate constants with relatively small errors.

When comparing the rate constants determined for both fully metalated and partially metalated Cd‐MT3, several observations can be made. In general, it appears that the last oxidation step (*k*_6_) where Cd_3_MT3 demetallates to Cd_0–2_MT3 is the fastest step for both fully and partially metalated Cd‐MT3. In addition, it appears that there is a noticeable decrease in the *k*_5_ (where Cd_4_MT3 is oxidized, resulting in Cd_3_MT3), pointing to some stability of the Cd_4_MT3 species. This was confirmed with the data in Fig. 5 where the Cd_4_ cluster was detected in the α domain. The trend in *k*_2–5_ for the fully metalated Cd_7_MT3 is shown in Fig. 6G, where there is a systematic reduction. However, *k*_1_ does not follow the trend of *k*_2–5_, as it is lower than *k*_2_. It is possible that the Cd_8+_MT3 to Cd_7_MT3 step (*k*_1_) is affected by the adducts that appear in the mass spectra, where we note that there is already oxidation detected from the mass differences even for these species. The fastest step is the last step of oxidation (*k*_6_), followed by *k*_2_, similar to the values reported for MT1 previously [42]. The explanation for the fast rate of *k*_2_ may be that the first metals released, likely in the β domain of the protein, are relatively exposed. This is in agreement with the reports of the β domain of MT3 in the literature, where it is deemed to be highly fluxional [32, 50]. The pattern of the rate constants is more straightforward for the partially metalated Cd‐MT3 oxidation pathway, where the oxidation occurs with an increased rate as demetallation occurs, whereby the cysteines are less and less protected from the H_2_O_2_. In comparison to MT1, the rate constants follow the same pattern for partially metalated Cd‐MT, where the rate increases with further demetallation due to oxidation [42]. This may be due to the lack of fully formed α and β domain Cd‐thiolate clusters; therefore, the release of metals is not domain‐dependent, but solely dependent on the exposure of cysteinyl thiols to the oxidizing agent.

### Oxidation pathway of partially metalated Zn‐MT3

**Fig. 7:**
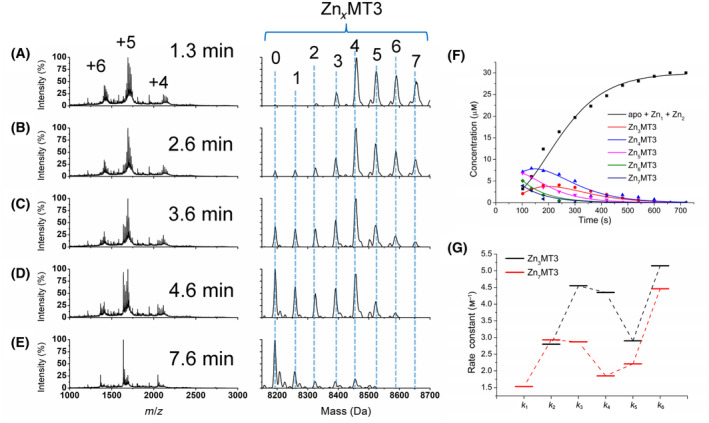
Reaction of Zn_3_MT3 with 150 molar equivalents of H_2_O_2_ at pH 7.4 monitored over time using ESI‐MS and UV–visible absorption spectroscopy. The concentration of Zn_3_MT3 used for ESI‐mass spectral analysis was 30 μm. (A–E) Selected mass spectral data of the reaction at time points (A) 1.3 min, (B), 2.6 min, (C) 3.6 min, (D), 4.6 min, and (E) 7.6 min. The charges state spectra are the left panels. The charge states labeled in Panel A apply to the panels below. The corresponding deconvoluted mass spectra are shown in the right panels. The masses for each Zn_*x*_MT3 species (*x* = 0–7) are labeled. Note that the peaks may not exactly match that calculated for the Zn_*x*_MT3 species due to addition of oxygen (+16 Da) as well as oxidation of cysteines to disulfide bonds. (F) Corresponding concentrations of each Zn_*x*_MT3 species as a function of time (points) extracted from the ESI‐mass spectral data with fitted speciation calculated by copasi (solid lines). (G) Rate constants (k) for each oxidation step outlined in Scheme 2 for Zn_3_MT3 (black) and Zn_7_MT3 (red). The data presented represents *n* = 1 of 3 repeated experiments.

Partially metalated Zn‐MT3 (Zn_3_MT3) oxidation was then investigated (Fig. 7). A lower concentration of Zn_3_MT3 (30 μm) was used due to the speed at which the reaction occurred; however, the 150 molar equivalents of H_2_O_2_ was kept constant to ensure continuity between all experiments reported. Figure 7A–E shows a selection of the charge state and deconvoluted mass spectra recorded, with the charge states labeled in Fig. 7A corresponding to all the panels below and the masses of the oxidized Zn‐MT3 species labeled in the deconvoluted spectrum. The charge state spectra are dominated by the +5 charge state at 1.3 min into the reaction and it continues through the oxidation reaction (Fig. 7A). The exposure of partially metalated Zn_3_MT3 to H_2_O_2_ initially causes additional Zn(II) to bind; however, it is likely due to adduct formation as postulated above for Zn_7_MT3. The oxidation then occurs rapidly, where apo‐MT3 is the dominant species within 7.6 min (Fig. 7E) even at a reduced concentration of Zn_3_MT3. Between 2.6 and 4.6 min of the reaction, it is clear that there is a retention of the Zn_4_MT3 species (Fig. 7B–D). We can propose that the Zn_4_ cluster in the α domain protects the protein from further rapid oxidation and is a relatively stable structure in comparison to the other Zn_*x*_MT3 species. Unlike the case with the Cd_4_ cluster, a clear derivative band representative of the symmetry of the Zn_4_ cluster was not noted in the CD spectrum (Fig. 5). The species are plotted as a function of time and fitted to rate constants using copasi software (Fig. 7F). The rate constants determined by copasi are as follows (*k*_2–6_): 2.9 ± 0.8 m ^−1^·s^−1^, 1.9 ± 0.8 m ^−1^·s^−1^, 2.0 ± 0.1 m ^−1^·s^−1^, 1.7 ± 0.1 m ^−1^·s^−1^, and 1.4 ± 0.5 m ^−1^·s^−1^. The rate constants of both partially and fully metalated Zn‐MT3 reaction with H_2_O_2_ are summarized in Fig. 7G.

When looking at the pattern of the rate constants for Zn‐MT3 oxidation, the fastest step is again the last step of oxidation (*k*_6_) where Zn_3_MT3 oxidizes to form Zn_0–2_MT3. We postulate that this is due to the exposure of the cysteinyl thiols to the oxidant, similar to the reaction of Cd‐MT3 oxidation. Similarly, *k*_5_ is slower than most other steps in the oxidation pathway, which is representative of the step where Zn_4_MT3 oxidizes to Zn_3_MT3. Due to the presence of Zn_4_MT3 cluster in the ESI‐mass spectral data, it is reasonable to conclude that the cluster imparts some stability and shielding from oxidation [32, 43].

The overall pattern of the rate constants for the oxidation of Zn‐MT3 is similar to those of Cd‐MT3. In the same way, there is a decrease in the rate constants *k*_2–4_ for fully metalated Zn‐MT3, which may be indicative of the way that fully metalated MT3 species follow a pathway that is more domain‐specific. We see the rapid release of the metals from the β domain with *k*_2_, and a lower *k*_6_ for the release of metals form the α domain, with decreasing rate constants in between possibly due to the movement of metals to a stable cluster. This contrasts with the partially metalated Zn‐MT3 species, where the rate constants overall increase from *k*_2–4_ with *k*_3_ and *k*_4_ with similar values. This is the pattern that is followed by partially metalated Cd‐MT3 as well and follows the interpretation of cluster‐independent oxidation of MT3 with the exception of the formation of the α domain cluster.

### Cysteine disulfide bond formation during the oxidative pathway of Zn‐MT3 and Cd‐MT3

**Fig. 8:**
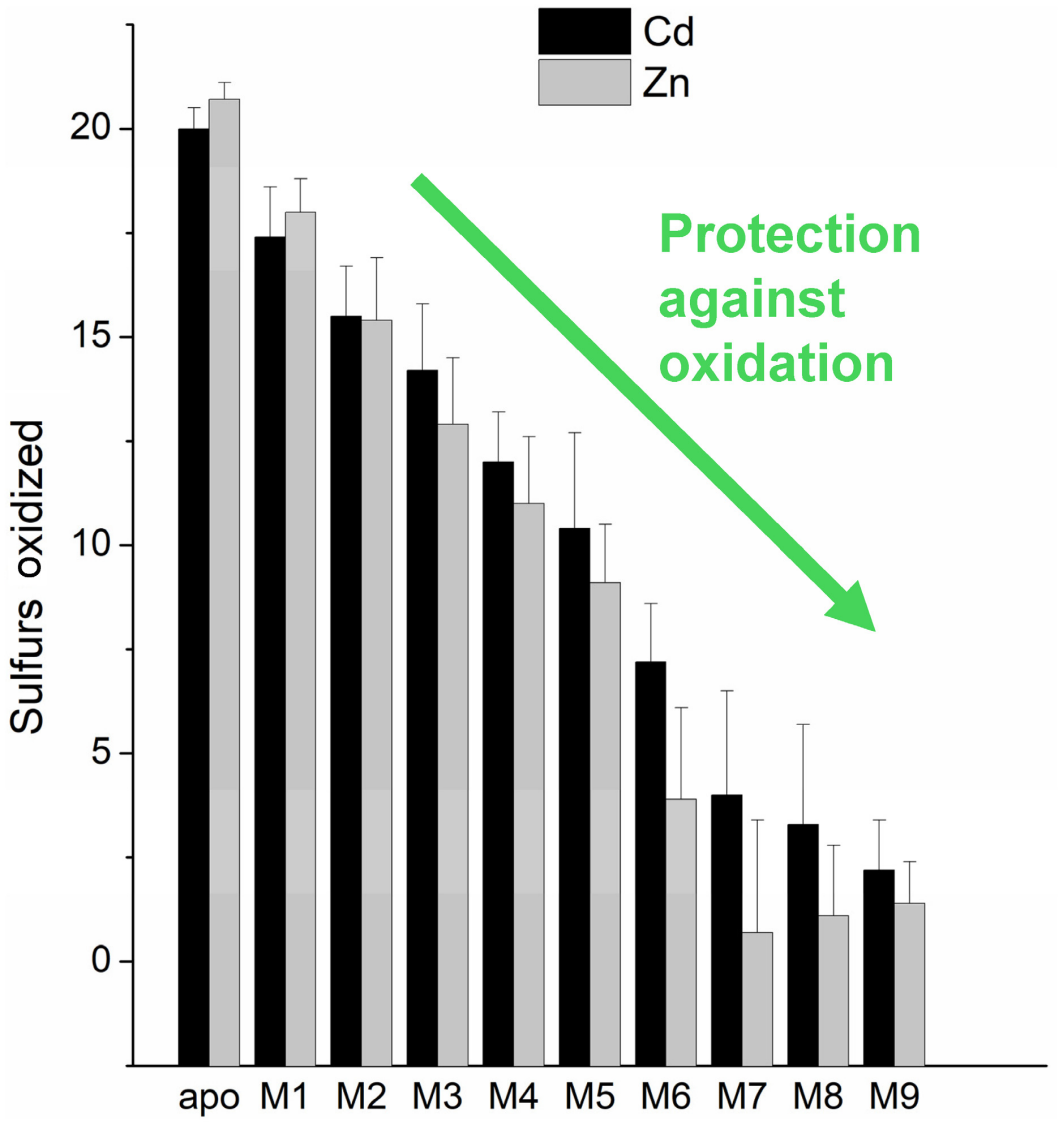
Number of sulfurs oxidized for each M_*x*_MT3 species (where M = Cd or Zn; *x* = 0–10) with standard deviations shown as error bars. These data were created through Tables 1 and 2, as well as Figs 3, 4, 6, and 7 (*n* = 3–6 depending on metalated species studied). The increase in metal loading serves as a protective mechanism against disulfide bond formation.

The numbers of oxidized cysteines from all the oxidation data collected were then analyzed and are summarized in Fig. 8. The numbers of cysteines oxidized for Zn‐MT3 and Cd‐MT3 are similar and not significantly different. In general, approximately 2–4 cysteines are oxidized in each step. It is important to note, however, that there is a large error in the number of cysteines oxidized in Cd/Zn_7–10_MT3 species, as the adduct formation is not uniform due to the lack of specificity of binding as these are not defined binding sites for MT3.

## Conclusions

We report ESI‐mass spectra, CD, and UV–visible absorption spectra detailing the pathway of oxidation of fully and partially metalated Zn/Cd‐MT3. We determined the rate constants for each step of the oxidation pathway, where metals are lost sequentially with the exception of the last two metal ions that are lost in one step. The pathways follow a domain‐specific pattern that can be explained by the dominance of the clusters that form in the α domain for fully metalated Cd/Zn_7_‐MT3. Specifically, the first metals from the β domain are lost at a rapid rate due to the exposure and fluxionality of the β domain and the Zn/Cd_4_ cluster in the α domain is retained for longer in the pathway and is fitted to a lower rate constant than expected. However, these pathways are significantly different for partially metalated Zn/Cd‐MT3, where the species representative of the clusters within each domain are less influential of the rates of oxidation. The fastest step in the oxidation reaction for all Zn/Cd‐MT3 species is the last step where Cd/Zn_3_MT3 is oxidized to form Cd/Zn_0–2_MT3; we believe this is when the protein is most exposed to the H_2_O_2_. These rates of oxidation are 5–10× faster than reported for MT1, suggesting the reactivity of MT3 with an oxidant is greater than that of MT1. Together, these data support the understanding of MT3 as a key player in control of reactive oxygen species in the cellular environment.

## Materials and methods

Materials and methods are based on those described in the paper by Yuan *et al*. [59] Briefly, they are described below.

### MT3 expression

Recombinant MT3 was cloned into pET29a plasmids with restriction enzymes Ncol and Hindll (GENEWIZ from Azenta, San Francisco, CA, USA). Competent BL21(DE3) *Escherichia coli* cells were transformed with the plasmid according to the manufacturer's instructions (Sigma Aldrich, St. Louis, MO, USA). The sequence of the recombinant MT3 was as follows: GSMGKAAA MDPETCPCPSGGSCT CADSCKCEGC KCTSCKKSCC SCCPAECEKC AKDCVCKGGE AAEAEAEKCS CCQKKAAAA. Detailed procedures used to express the MT3 were detailed in Yuan *et al*. [52] Briefly, cells were grown to OD_600_ before MT3 expression was induced by isopropyl β‐d‐thiogalactoside (IPTG; BioShop, Toronto, ON, Canada) and stabilized with CdSO_4_. Cells were grown for an additional 3.5 h before pelleting through centrifugation (Avanti J‐26 XPI fixed rotor centrifuge; Beckman Coulter, Toronto, ON, Canada) and suspension in 20 mm tris‐hydroxymethyl‐aminomethane hydrochloride (Tris/HCl) buffer (Sigma Aldrich) at pH 7.4.

### MT3 purification

Cells were lysed using a cell homogenizer (Constant Systems, Hull, UK) and treated with bovine DNAse (Sigma Aldrich) following the manufacturer's instructions. The lysate was then isolated and loaded on both anion and cation exchange columns before separation using high‐pressure liquid chromatography (Dionex UltiMate 3000, Thermo Fisher, Toronto, ON, Canada). The detailed protocol is outlined in Yuan *et al*. [52].

### MT3 for ESI‐MS preparation

MT3 was thawed and demetalated with dearated and argon‐saturated pH 2 ammonium formate solution and buffer‐exchanged using Amicon Ultra‐4 centrifugal tubes (5 kSa MWCO; Millipore, Burlington, VT, USA). Apo‐MT3 was then desalted and pH‐adjusted with buffer exchange with 20 mm Tris/HCl with 0.5 mm tris(2‐carboxyethyl)phosphine (TCEP)‐HCl (Soltec Ventures, Beverly, MA, USA). Concentrations of MT3 were determined using ε250 nm = 89 000 m ^−1^·cm− in the presence of excess Cd(OAc)_2_.

### ESI‐MS procedures

All ESI‐mass spectra were collected using a Bruker Micro‐TOF II spectrometer (Bruker Daltonics, Toronto, ON, Canada) and calibrated using the Bruker calibration mix on positive ion mode. The settings are as follows: scan = 1000–3000 *m/z*; rolling average = 2; nebulizer = 2 bar; dry gas = 130 °C at 6 L·min^−1^; capillary = 3500 V; end plate offset = −500 V; capillary exit = 175 V; skimmer 1 = 30.0 V; skimmer 2 = 23.5 V; hexapole RF = 800 V. Spectra were collected for 2 min and deconvoluted with the Bruker compass data analysis software on maximum entropy mode.

For kinetic experiments, three replicates were measured to ensure accuracy. The temperature of the syringe pump was recorded to be 304 K constantly at ambient temperature.

### Solution preparation

Hydrogen peroxide was prepared following the methods outlined in detail by Korkola and Stillman [42]. In short, hydrogen peroxide was diluted with dearated distilled water before addition to protein samples.

Cd(OAc)_2_ (Acros Organics, Toronto, ON, Canada) was prepared as a 10 mm solution by dissolving in deionized water.

Zn(OAc)_2_ (Fisher Scientific, Toronto, ON, Canada) was prepared as a 10 mm solution by dissolving in deionized water.

### UV–visible and circular dichroism spectroscopy procedures

UV–visible absorption data were recorded on a Cary 60 spectrophotometer (Agilent, Santa Clara, CA, USA). All circular dichroism (CD) spectra were measured on a Jasco J810 with and averaged over two accumulations (Easton, MD, USA). Spectra were measured over a range of 200–300 nm with an air baseline and subtracted buffer baseline. A 1‐cm path length cuvette was used for all experiments on the Cary 60 (Hellma Analytics, Plainview, NY, USA), and a 0.2 cm path length cuvette was used for circular dichroism experiments (Hellma).

### copasi setup

For the fitting of kinetic constants, copasi fitting program was used. The bimolecular reactions were defined as (where [*X*] is the initial concentration):

$$M_{n-1}\mathrm{MT}3+M\;\overset{k_{n}}{\to }M_{n}\mathrm{MT}3,$$

where *k*_*n*_ is the rate constant and then

$$\text{Rate}=k_{n}\left[M\right]\left[M_{n-1}\mathrm{MT}3\right].$$

The fitted rate constants (*k*_*n*_) were calculated by the copasi program using a least square minimization method when given the speciation data obtained experimentally.

## Conflict of interest

The authors declare no conflict of interest.

## Author contributions

ATY and MJS planned the experiments, and ATY carried out all the experimental work. Both authors wrote and approved the final manuscript.

## Data Availability

The data that support the findings of this study are available in all figures. Detailed spreadsheets of data are available upon request.
